# Angiotensin II status and sympathetic activation among hypertensive patients in Uganda: a cross-sectional study

**DOI:** 10.1186/s13104-015-1544-7

**Published:** 2015-10-20

**Authors:** Jonathan Mayito, Michael Mungoma, Barbara Kakande, Dove Clement Okello, Humphrey Wanzira, James Kayima, Charles Kiiza Mondo

**Affiliations:** Department of Medicine, College of Health Sciences, Makerere University, P.O. Box 7072, Kampala, Uganda; Department of Medicine, Mulago Hospital, Kampala, Uganda; Uganda Heart Institute, Kampala, Uganda; Infectious Diseases Research Collaboration, Kampala, Uganda; Non Communicable Disease Alliance, Kampala, Uganda

**Keywords:** Angiotensin II status, Sympathetic nervous activity, Hypertension

## Abstract

**Background:**

Sympathetic activation and renin-angiotensin system are essential for development and sustenance of hypertension. However, the status of these systems has not been well evaluated among patients in an African setting. This study therefore set out to assess the angiotensin II status and sympathetic activation among hypertensive patients in Uganda.

**Methods:**

In this cross sectional study conducted at Mulago, the national referral hospital, blood samples were taken to measure angiotensin II, metanephrines and normetanephrines. Urine samples were also taken for measuring urine creatinine and sodium. The angiotensin II categories were defined using the Mosby’s Diagnostic and Laboratory Test References. 9th ed while the metanephrines and normetanephrine categories were defined using the Makerere University Biosafety II Immunology Laboratory reference values.

**Results:**

162 patients were consented and enrolled into the study, of these 136 (84 %) had low, 15 (9 %) had normal, while, 11 (7 %) had high angiotensin II levels. 142 (88 %) participants had normal levels of metanephrine, while 20 (12 %) had high levels. Only 88 were assessed for metanephrines and of these 85 (97 %) had normal, while 3 (3 %) had raised levels. Urine sodium was associated with low and normal angiotensin II levels (P value 0.007). Female gender and diastolic blood pressure were associated with a protective effect against high normetanephrines (OR 0.29, P value 0.015), 80–89 mmHg (OR 0.19, p value 0.053), above 100 mmHg (OR 0.27, p value 0.022). Current smoking status was associated with high risk for abnormal normetanephrines (OR 17.6, P value −0.022) while former smoking was associated with high risk for abnormal metanephrines (OR 18.7, p value 0.022). After multivariate analysis, all the significant variables at bivariate analysis were still significant except those who stopped smoking and those with a BP at 80–89 which were not significant.

**Conclusions:**

Hypertensive patients in this setting have predominantly low angiotensin II hypertension as a result of high salt intake. Sympathetic activation is not a significant mechanism of hypertension in this study population, more so in the females, with the exception of smokers who have a highly activated sympathetic system. Therefore, the use of agents targeting renin angiotensin and sympathetic systems as single first line antihypertensive agents in this setting should be re-evaluated if such patients are to be treated effectively.

## Background

Hypertension is one of the most prevalent and major contributors to atherosclerotic cerebral and cardiovascular disease, increasing the risk by two to threefold [1]. Treatment of hypertension is associated with decline in the risk of stroke (30–40 %), coronary artery disease (20 %) and other major cardiovascular diseases (21–28 %) [2].

Hypertension is a result of the interaction between genetic and environmental factors. This interaction influences intermediary phenotypes such as sympathetic nervous activity, renin angiotensin aldosterone, renin-kallikrein—kinin systems, and endothelial factors [3]. These phenotypes in turn influence other intermediary phenotypes such as sodium excretion, vascular reactivity, and cardiac contractility, which, determine total vascular resistance and cardiac output, and therefore blood pressure [3].

More than 70 % of hypertensive patients have renin related mechanisms as the aetiology of their hypertension. About 20 % of these have inappropriately normal or high renin values, and 30 % have low renin values, with the remaining half distributed between these two extremes [4]. Angiotensin II on the other hand has been shown to cause hypertension and vasculopathy through the activation of the mitogen-activated protein (MAP) kinase activity which mediates vascular smooth muscle proliferation [5] The effects of angiotensin II are compartmentalised mainly in the medulla and tubule of the kidney, where it regulates medulla and tubular function through its type 1 receptor [6]. The Na^+^/H^+^ exchanger 3 in the proximal tubule is also key in maintaining basal blood pressure and the development of angiotensin II hypertension [7], a phenomena that should be of interest in blacks that predominantly have salt sensitive and low renin hypertension compared to the white population [8].

Sympathetic neural mechanisms are also important in the development and progression of hypertension. The magnitude of sympathetic activation is proportional to the degree of elevation in blood pressure and development of hypertension-related target organ damage [9]. It has been suggested that repeated stress-induced sympathetic activation contributes to the pathophysiology of hypertension in blacks unlike in white populations [10, 11]. Increased serum and urinary metanephrines and normetanephrines are a measure of sympathetic activation and are used in the diagnosis of pheochromocytoma [12, 13].

Much has been studied about the renin angiotensin and sympathetic nervous systems’ role in the pathophysiology of hypertension. Despite this, data on this subject in an African setting is limited. The closest data often cited for black populations is from the African Americans who may be genetically and environmentally distinct from blacks in an African setting. There is a need to fill this knowledge gap with data from an appropriate population. We therefore set out to assess the angiotensin II status and sympathetic activation of hypertensive patients in Uganda attending the national referral hospital. Such information is extremely useful, especially in our low resource settings, where appropriate treatment of chronic conditions, basing on scientific evidence, is prudent. We also sought to seek for any factors associated with the prevailing status of these parameters.

## Methods

This was a cross sectional study conducted at Mulago, the only National referral hospital in Uganda.

All study participants were recruited from the Mulago Hospital hypertension clinic. We recruited both newly diagnosed adult hypertensive patients (with no history of antihypertensive medication) and previously treated hypertensive patients but who had defaulted their medication for at least 1 week. Exclusion criteria included; pregnant women, patients currently or within 1 week of using oral contraceptive therapy or adrenaline, patients with deranged renal function tests and urinalysis, patients with diabetes mellitus and confirmed pheochromocytoma.

A formula by Eng [14] was used to estimate the sample size. A total of 162 respondents were computed basing on 95 % confidence interval, a precision of 5 and 10 % of the sample size used to compensate for non-respondents.

The study participants were consecutively recruited from the waiting area in the hypertension clinic and screened using the study eligibility criteria. Those eligible were informed about the study and requested to give a written informed consent to participate in the study.

Study participants responded to a pre-coded, pre-tested and standardized questionnaire which covered demographic details, duration of hypertension, duration off antihypertensives, type of antihypertensives that were being taken before defaulting, alcohol consumption, salt intake and smoking. They then underwent measurement of height, weight and blood pressure, and then gave an arterial blood and urine sample as elaborated below.

The body mass index (BMI) was calculated using the formula; weight (kg)/height (m^2^) and then categorized into underweight (<18.5), Normal weight (18.5–24.9), overweight (25–29.9) and obese (>30) using the world health organisation criteria of categorization of BMI, 2004 [15].

The blood pressure was measured on the left arm after the subject had sat for at least 10 min, using an Omron M7 (HEM-780-E) oscillometric blood pressure monitoring sphygmomanometer with the subject in the sitting position, legs uncrossed, the arm resting on a table and the ante-cubital fossa at the level of the lower sternum. The Omron M7 (HEM-780-E) is validated according to the British hypertension Society protocol and is recommended for professional and home use [16].

An appropriate cuff (with bladder length >80 % of the arm circumference) was used. Two readings were taken 3 min apart and the average was used to describe the blood pressure of the patient. If the readings differed by 10 mmHg, a third reading was taken and the blood pressure was then taken to be the average of the closest two. Blood pressure was then categorized using the JNC 7 [17].

The participant was laid on the examination couch in supine position for at least 15 min before the blood sample was drawn. Using a 10 ml syringe and observing aseptic conditions, 6 ml of blood was drawn from the femoral artery. Pressure was applied to the puncture site for 7 min to stop any bleeding. Four ml was introduced into an iced pre-labelled EDTA vacutainer, mixed gently by tilting the vacutainer top to bottom and vice versa eight times to mix the blood with the anticoagulant.

The sample was kept under ice in an ice box carrier immediately. It was then transported within 1 h to the laboratory where it was centrifuged at 4500 rotations per minute at 4 °C for 5 min to separate the plasma. The plasma was stored at −80 °C till analysis. The remaining ml were used for determination of plasma creatinine and sodium at the Mulago Hospital clinical chemistry laboratory.

All the samples were analysed within 5 months from the time of collection at the Makerere University Biosafety II Immunology Laboratory, using the AssayMax Human Angiotensin II Elisa Kit from ASSAYPRO, Germany and 2-MET Plasma Elisa fast track from Labor Diagnostic Nord, Germany.

The Mosby’s Diagnostic and Laboratory Test References. 9th ed angiotensin II reference ranges (0.01–0.06 ng/ml) and the Makerere University Biosafety II Immunology Laboratory reference values for metanephrine (<90 pg/ml) and normetanephrine (<180 pg/ml) were used to categorise these measurements into low, normal, and high [18, 19].

Patients were instructed to collect a mid-stream urine sample after washing with soap the head of the penis and the retracted foreskin (for men) or the separated skin folds covering the urinary opening (for females). The urine sample was transported within 1 h to the laboratory for determination of urine sodium and creatinine.

### Data analysis

Data analysis was done with the assistance of a statistician. Data were double entered using EPI-INFO 6.0 and then exported to STATA version 12.0 (StataIC Corporation, College Station, TX, USA) for analysis.

The independent variables which included: social demographics, alcohol intake, smoking history, salt intake, treatment history of hypertension, physical measurements, and fraction excretion of sodium were organised into categories. The outcomes of interest in this study were percentages and their confidence intervals of the participants with low, moderate and high angiotensin II, metanephrine and normetanephrines levels.

Logistics regression model was used to assess for factors associated with the three parameters outcomes by estimating the odds ratio and accompanying 95 % confidence interval. Only variables that were significant in the bivariate analysis (gender, smoking and diastolic BP) were considered for multivariate analysis, and a forward fitting regression model was used to assess for effect modification and confounders. In all analyses, a *P* value of ≤0.05 was considered to be statistically significant.

### Supporting data

The full dataset for this study is available on Zenodo data repository. DOI 10.5281/Zenodo.31479.

### Ethical approval

Written informed consent was obtained from all study participants. The study protocol was approved by the Makerere University School of Medicine Research and Ethics Committee, and Uganda National Council of Science and Technology.

## Results

**Fig. 1:**
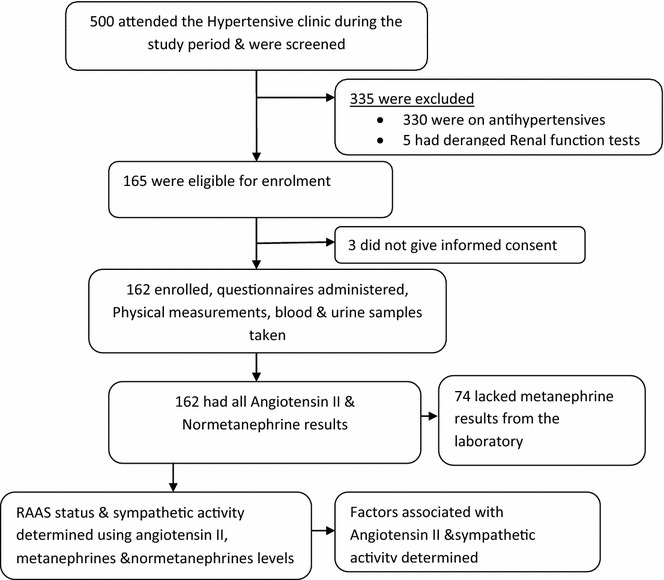
Flow chart for participants’ selection

162 Patients were recruited for the study as shown in Fig. 1. Majority of the participants were female, 131 (81 %), and Baganda were the most represented ethnic group, 116 (71.6 %).

There were more senior citizens, 122 (75 %), above 45 years and 61 (38 %) above 60 years with hypertension compared to the younger participants 40 (25 %) below 45 years and 6 (4 %) participants below 30 years (Table 1).

**Table 1:** Baseline characteristics

Characteristic	Study participants	
	Number, total = 162	%
Age in years		
≤45	40	25
>45	122	75
Age distribution		
18–40	25	15
41–60	76	47
>60	61	38
Gender		
Male	31	19
Female	131	81
Tribe		
Buganda	116	72
Basoga	7	4
Banyankole	13	8
Banyoro	5	3
Others	21	23
Occupation		
Peasant/farmer	69	43
Manual labourer	50	31
Office worker	7	4
Unemployed	36	22
Level of education		
No formal	22	14
Primary	96	59
Secondary	37	23
Tertiary	7	4
Marital status		
Single	11	7
Married	80	49
Divorced/separated	71	44

Sixty-seven (42 %) of the participant added raw salt to their food. Among the 162 participants, majority 64 (40 %) of them added one table spoon to their food as they prepared it, closely followed by 54 (33 %) who added half a table spoon.

Majority of the participants, 136 (84 %) had normal pulse rates and majority had a systolic blood pressure of more than 160 mmHg and a diastolic blood pressure of more than 100 mmHg, 108 (67 %) and 89 (55 %) respectively. Twenty-nine (18 %) participants had grade one obesity while 9 (6 %) were morbidly obese. See Table 2.

**Table 2:** Physical measurements

Characteristic	Study participant	
	Number, total = 162	%
Pulse rate		
<60	8	5
61–100	136	84
>100	18	11
Systolic blood pressure		
<120	3	2
120–139	12	7
140–159	39	24
>160	108	67
Diastolic blood pressure		
<80	22	14
80–89	25	15
90–99	26	16
≥100	89	55
BMI		
<18.5	9	6
18.5–24.9	63	39
25–29.9	51	32
30–34.9	29	18
>35	9	6

Sixteen (10 %) participants were recently diagnosed with hypertension compared to 146 (90 %) who had a known diagnosis of hypertension but had defaulted taking their medications. Among the 146 previously treated defaulting participants, 34 (23 %) of them had been on single drug therapy while 112 (77 %) had been on combination therapy. The most commonly used class of single drug therapy was calcium channel blockers, 13 (38 %) while the most commonly used combination therapy was a diuretic with a calcium channel blocker and either an ACEI or ARB 27 (24 %). See Table 3.

**Table 3:** Treatment history

Characteristic	Study participant	
	Number, total = 146	%
Time off antihypertensives		
1 week	50	34
1 to <2 weeks	42	29
2 to ≤4 weeks	17	12
1 month	13	9
>1 month	22	15
No record	2	1
Single drug antihypertensives		
Duiretic	2	6
Calcium channel blocker	13	38
Beta blocker	8	24
ACEI/ARBs	9	26
Others	1	3
No record	1	3
Combination drug antihypertensive		
Duiretic and Calcium Ch.	13	12
Duiretic and ACEI/ARB	5	4
Calcium Ch and beta blocker	10	9
Calcium Ch. and ACEI/ARB	19	17
Beta block + ACEI/ARBs	6	5
Duiretic + calcium ch + ACEI/ARB	27	24
Others	27	24

The smoking rates in this study were very low, with 3 (1.9 %) and 12 (7 %) being current and former smokers respectively. The participants currently taking alcohol were 35 (22 %). Among the 35 participants, 18 (51 %) were taking beer, followed by local gin (waragi) at 12 (34 %). Among the 31 who took quantifiable amounts of alcohol, the majority took 1–6 bottles of beer per week while only one participant took a glass of wine daily.

## Renin angiotensin status

Eighty-four percent (136) of the participants had low angiotensin II levels. This represented a proportion of 78–90 % of the reference population as shown by the 95 % confidence interval. Among the participants with low angiotensin II levels, 130 (97 %) had normal, 4 (3 %) had low while none had high urine excretion of sodium. In contrast however, majority with low angiotensin II levels, 120 (90 %), had a fractional excretion of sodium of less than 1 %. Urine sodium was the only factor significantly associated with low and normal angiotensin II levels, P value = 0.007 as illustrated in Table 4.

**Table 4:** Bivariate for angiotensin II levels and associated factors

Risk factor	Angiotensin II categories			
	<0.01 (low)	0.01–0.06 (normal)	>0.06 (high)	P value
	Number (%)	Number (%)	Number (%)	
Age in years				
≤45	32 (23.53)	6 (40.00)	2 (18.18)	0.326
>45	104 (76.47)	9 (60.00)	9 (81.82)	
Gender				
Male	25 (18.38)	5 (33.33)	1 (9.09)	0.257
Female	111 (81.62)	10 (66.67)	10 (90.91)	
Smoking				
No	2 (1.47)	0	1 (9.09)	0.111
Stopped	126 (92.65)	12 (80.00)	9 (81.82)	
Yes	8 (5.88)	3 (20.00)	1 (9.09)	
Alcohol				
Yes	30 (22.22)	2 (13.33)	3 (27.27)	0.657
No	105 (77.78)	13 (86.67)	13 (86.67)	
Systolic BP				
<120	3 (2.21)	0	0	0.930
120–139	10 (7.35)	1 (6.67)	1 (9.09)	
140–159	34 (25.00)	2 (13.33)	3 (27.27)	
>160	89 (65.44)	12 (80.00)	7 (63.64)	
Diastolic BP				
<80	22 (16.18)	0	0	0.221
80–89	19 (13.97)	4 (26.67)	2 (18.18)	
90–99	24 (17.65)	1 (6.67)	1 (9.09)	
≥100	71 (52.21)	10 (66.67)	8 (72.73)	
Pulse rate				
<60	8 (5.88)	0	0	0.373
61–100	112 (82.35)	15 (100)	9 (81.82)	
>100	16 (11.76)	0	2 (18.18)	
Salt intake				
Yes	56 (41.18)	7 (46.67)	4 (36.36)	0.865
No	80 (58.82)	8 (53.33)	7 (63.64)	
Urine sodium (mmol/l)				
<20	4(2.99)	3 (20.00)	0	*0.007*
20–350	130 (97.01)	12 (80.00)	11 (100)	
FENa				
<1	120 (89.55)	14 (93.33)	7 (70.00)	*0.059*
1–2	9 (6.72)	0	3 (30.00)	
>2	5 (3.73)	1 (6.67)	0	
Time since diagnosis				
<1 month	11 (8.09)	3 (20.00)	2 (18.18)	0.697
1 to <6 months	8 (5.88)	1 (6.67)	0	
6 to <12 months	9 (6.62)	1 (6.67)	1 (9.09)	
>1 year	108 (79.41)	10 (66.67)	8 (72.73)	
Time off antihypertensives				
1 week	45 (36.59)	2 (15.38)	3 (37.50)	0.360
1 to <2 weeks	34 (27.64)	7 (53.35)	1 (12.50)	
2 to ≤4 weeks	14 (11.38)	1 (7.69)	2 (25.00)	
1 month	11 (8.94)	2 (15.38)	0	
>1 month	19 (15.45)	1 (7.69)	2 (25.00)	

*FENa* fraction excretion of urine sodium

The p values in italics indicate factors associated with angiotensin II at α = 0.05

## Sympathetic nervous activity

Majority of the participants, 142 (88 %) had normal normetanephrine levels representing a range of 83–93 % in the reference population as shown by the 95 % confidence intervals. A similar proportion, 85 of the 88 (97 %) participants with metanephrine results had normal metanephrine levels representing 92–100 % of the reference population as shown by the 95 % confidence intervals.

Among the participants with normal metanephrines, 60 (71 %) had a systolic blood pressure of more than 160 mmHg compared to 94 (66 %) with normal normetanephrines. The percentage of participants with normal metanephrines and normetanephrines who had a diastolic blood pressure of more than 100 mmHg was similar, 55 and 56 % respectively.

Distribution by the other variable is shown in Table 5.

**Table 5:** Distribution of metanephrines and normetanephrines by different variables

Characteristic	Metanephrines		Normetanephrines	
	Normal, N = 85 (%)	High, N = 3 (%)	Normal, N = 142 (%)	High, 20 (12 %)
Gender				
Male	21 (25)	2 (67)	23 (16)	8 (40)
Females	64 (75)	1 (33)	119 (84)	12 (60)
Age				
<45	24 (28)	2 (67)	36 (25)	4 (25)
≥45	61 (72)	1 (33)	106 (75)	16 (75)
Systolic BP				
<140	5 (6)	0	14 (10)	1 (5)
140–160	20 (24)	1 (33)	34 (24)	5 (25)
>160	60 (71)	2 (67)	94 (66)	14 (70)
Diastolic BP				
<90	23 (27)	1 (33)	38 (27)	9 (45)
90–100	7 (8)	0	25 (18)	1 (5)
>100	55 (65)	2 (67)	79 (56)	10 (50)
Pulse				
<60	6 (7)		7 (5)	1 (5)
60–100	67 (79)		119 (84)	17 (85)
>100	12 (14)		16 (11)	2 (10)

Being female was associated with a significant protective effect from high normetanephrine OR 0.29 (0.11–0.79), P = 0.015 and so was diastolic blood pressure of 80–89 mmHg OR 0.19 (0.03–1.02), p = 0.053, 90–99 mmHg OR 0.86 (0.01–0.77), P = 0.028 and >100 mmHg OR 0.27 (0.83–0.89, p = 0.022, as shown in Table 6.

**Table 6:** Bivariate analysis for factors associated with metanephrines and normetanephrines

Risk factor	Metanephrines			Normetanephrines		
	Odds (95 % CI)	OR (95 % CI)	p value	Odds (95 % CI)	OR (95 % CI)	p value
Age in years						
≤45	0.08 (0.02–0.35)			0.11 (0.04–0.31)		
>45	0.02 (0.00–0.12)	0.20 (0.02–2.37)	0.154	0.15 (0.08–0.26)	1.4 (0.43–4.33)	0.604
Gender						
Male	0.10 (0.02–0.41)			0.35 (0.16–0.78)		
Female	0.02 (0.00–0.11)	0.16 (0.01–2.00)	0.106	0.10 (0.06–0.18)	0.29 (0.11–0.79)	*0.015*
Smoking						
No	0.01 (0.00–0.10)	Reference		0.11 (0.07–0.19)	Reference	
Stopped	0.25 (0.05–1.18)	18.75 (1.53–230.42)	*0.022*	0.33 (0.09–1.23)	2.93 (0.72–12.03)	0.135
Yes	0	–	–	2.00 (0.18–22.06)	17.6 (1.50–205.82)	*0.022*
Alcohol						
Yes	0.11 (0.03–0.48)			0.21 (0.09–0.50)		
No	0.02 (0.00–0.11)	0.14 (0.01–1.69)	0.069	0.13 (0.07–0.22)	0.60 (0.21–1.71)	0.342
Systolic BP						
<120	0			0.50 (0.05–5.51)	Reference	
120–139	0			0	–	–
140–159	0.05 (0.01–0.37)	Reference		0.15 (0.06–0.38)	0.29 (0.02–3.87)	0.352
>160	0.03 (0.01–0.14)	0.67 (0.06–7.88)	0.746	0.15 (0.08–0.26)	0.30 (0.03–3.50)	0.336
Diastolic BP						
<80	0.10 (0.01–0.78)	Reference		0.47 (0.19–1.14)	Reference	
80–89	–	–	–	0.087 (0.02–0.37)	0.19 (0.03–1.02)	*0.053*
90–99	–	–	–	0.04 (0.01–0.30)	0.86 (0.01–0.77)	*0.028*
≥100	0.04 (0.01–0.15)	0.36 (0.03–4.40)	0.426	0.12 (0.07–0.24)	0.27 (0.89–0.83)	*0.022*
Pulse rate						
<60	0	–	–	0.14 (0.02–1.16)	Reference	
61–100	0.04 (0.01–0.14)	–	–	0.14 (0.09–0.24)	1 (0.12–8.64)	1.00
>100	0	–	–	0.13 (0.03–0.54)	0.88 (0.07–11.31)	0.919
Salt intake						
Yes	0.07 (0.02–0.29)			0.16 (0.08–0.31)		
No	0.02 (0.00–0.13)	0.26 (0.02–3.08)	0.249	0.07 (0.07–0.25)	0.84 (0.33–2.17)	0.724
FENa						
<1	0.04 (0.01–0.13)			0.13 (0.08–0.22)	Reference	
1–2	0	–	–	0.20 (0.04–0.91)	1.56 (0.31–7.77)	0.586
>2	0	–	–	0.20 (0.02–1.71)	1.56 (0.17–14.23)	0.692
Time since diagnosis						
<1 month	0	Reference		0.07 (0.01–0.50)	Reference	
1 to <6 months	0.17 (0.02–1.38)	0.86 (0.04–18.73)	0.922	0.50 (0.13–2.00)	7.50 (0.65–87.19)	0.107
6 to <12 months	0.14 (0.01–1.16)	0.10 (0.00–1.97)	0.060	0.22 (0.05–1.03)	3.33 (0.26–42.21)	0.353
>1 year	0.02 (0.00–0.12)			0.13 (0.07–0.22)	1.88 (0.23–15.30)	0.557

*FENa* fractional excretion of sodium. Logistic regression model was used to determine the differences between the metanephrine and normetanephrine categories

The p values in italics indicate factors associated with metanephrines and normetanephrines at α = 0.05

Current smoking status was associated with a significantly increased risk of abnormal normetanephrine levels OR 17.6 (1.50–205.820), P value −0.022, while former smoking status was associated with increased risk of elevated metanephrines OR 18.75 (1.53–230.42), P = 0.022, as shown in Table 6.

After multivariate analysis, all the significant variables at bivariate analysis were still significant except those who stopped smoking and those with a BP at 80–89 which were not significant (Table 7).

**Table 7:** Multivariate analysis for associated factors normetanephrines

Normetanephrine	Adjusted OR (95 % CI)^a^	p value
Gender		
Male		
Female	0.25 (0.08–0.77)	0.016
Smoking		
No	Reference	
Stopped	2.16 (0.47–9.96)	0.323
Yes	21.03 (1.52–290.63)	0.023
Diastolic BP		
<80	Reference	
80–89	0.18 (0.03–1.10)	0.063
90–99	0.10 (0.01–0.98)	0.048
≥100	0.22 (0.06–0.73)	0.014

^a^Adjusted for gender, smoking and diastolic BP

## Discussion

The major finding in this study was that majority of the participants had low angiotensin II levels, which, correlated with the finding that majority of participants had a fractional excretion of sodium of less than 1 %. This clinical state is similar to patients with pre-renal azotemia whereby they are highly conserving sodium and water leading to a high effective circulatory volume. It is possible that high salt intake in this population suppresses angiotensin II release as majority of the participants reported taking salt in their diet, especially raw salt and urine sodium was associated with low angiotensin II levels. It would have been important to correlate the angiotensin II levels with renin levels as previous studies have shown majority of blacks to have a low renin hypertension [8] as a result of negative feedback from angiotensin II in a form of apparent minero-corticoid excess [20]. A full evaluation of the renin aldosterone angiotensin axis would have enabled more concrete conclusions. The fact that blacks are more responsive to diuretics and that addition of a diuretic improves efficacy of other antihypertensives in black populations unlike in white populations [21], further shows that salt plays a major role in the mechanism of hypertension in blacks. Other syndromes associated with conservation of sodium and water include; increased endothelin-1 activity [22] or a mutation in the epithelial sodium receptor (ENaC) [23] and these would require further evaluation in this study.

Findings from this study suggest that sympathetic nervous activation may not be a dominant mechanism in the pathophysiology of hypertension in this study population. This is in contrast to previous studies that have shown increased sympathetic nervous out flow in patients with accelerated or malignant hypertension, where, the sympathetic out flow is due to the increased endogenous renin-angiotensin axis which stimulates it at the sympathetic ganglia [24] and centrally [25]. These findings are also at variance with the suggestions that repeated stress-induced sympathetic activation initiates a cycle of increased vascular resistance and vascular hypertrophy leading to hypertension in black populations [10, 11]. We however, acknowledge that the smaller number of samples analysed for metanephrines could have under powered this study for making conclusions about the sympathetic activity. These results support the fact that beta blockers are not effective first line antihypertensive therapy, especially in black population and should therefore be reserved for compelling situations or improved by addition of a diuretic [26]. Furthermore, female gender was associated with a protective effect against high normetanephrines. This finding concurs with earlier findings which showed that autonomic blood pressure support is blunted in females more so in young women [27]. This attenuation of the sympathetic nervous system in females may be due to dampened sympatho-adrenal stimulation or augmented sympatho-adrenal inhibition [28].

More to the above, current smoking status was associated with increased stimulation of the sympathetic nervous activation meaning that smoking may be a contributing mechanism to developing hypertension in smokers. This finding is similar to results of other studies which showed that smoking has a direct peripheral and a centrally mediated effect on both blood pressure and pulse through stimulation of the sympathetic nervous system [29]. However, we acknowledge that the number of smokers was very low and this could have led to an over effect in assessing the associations.

It was also seen in this study that increase in diastolic blood pressure showed dampening or protection against increased sympathetic nervous activity. The diastolic blood pressure is related to relaxation of the cardiac muscle which occurs with reduced sympathetic outflow and sustained by residual pressure retained by the elasticity of the arterial system [30]. The sympathetic nervous system may therefore not play a significant role in diastolic hypertension in this study population.

## Conclusions

Hypertensive patients in this setting have predominantly low angiotensin II hypertension as a result of high salt intake. Sympathetic nervous activation is not a significant mechanism of hypertension in patients in this setting, more so in the females, but may be exaggerated in current smokers. Use of agents targeting renin angiotensin and sympathetic systems as single first line antihypertensive agents in this setting needs to be re-evaluated for better management of patients in this setting.
